# Ferroptosis in the Ovarian Follicular Microenvironment: A Redox-Dependent Cell Death Pathway with Emerging Roles in PCOS, Oocyte Quality, and IVF Outcomes

**DOI:** 10.3390/ijms262110381

**Published:** 2025-10-25

**Authors:** Charalampos Voros, Fotios Chatzinikolaou, Georgios Papadimas, Spyridon Polykalas, Despoina Mavrogianni, Aristotelis-Marios Koulakmanidis, Diamantis Athanasiou, Vasiliki Kanaka, Maria Kanaka, Kyriakos Bananis, Antonia Athanasiou, Aikaterini Athanasiou, Ioannis K. Papapanagiotou, Dimitrios Vaitsis, Charalampos Tsimpoukelis, Maria Anastasia Daskalaki, Marianna Theodora, Nikolaos Thomakos, Panagiotis Antsaklis, Dimitrios Loutradis, Georgios Daskalakis

**Affiliations:** 1 Department of Obstetrics and Gynecology, ‘Alexandra’ General Hospital, National and Kapodistrian University of Athens, 80 Vasilissis Sofias Avenue, 11528 Athens, Greece; depy.mavrogianni@yahoo.com (D.M.); aristoteliskoulak@gmail.com (A.-M.K.); vasilikikanaka@outlook.com (V.K.); maira.kanaka24@gmail.com (M.K.); tsimpoukelischa@gmail.com (C.T.); md181341@students.euc.ac.cy (M.A.D.); martheodr@gmail.com (M.T.); thomakir@hotmail.com (N.T.); panosant@gmail.com (P.A.); gdaskalakis@yahoo.com (G.D.); 2 Laboratory of Forensic Medicine and Toxicology, School of Medicine, Aristotle University of Thessaloniki, 54124 Thessaloniki, Greece; fotischatzin@auth.gr (F.C.); loutradi@otenet.gr (D.L.); 3 Athens Medical School, National and Kapodistrian University of Athens, 15772 Athens, Greece; dr.georgepapadimas@gmail.com (G.P.); spyridonpolykalas@hotmail.com (S.P.); gpapamd@hotmail.com (I.K.P.); vaitsisdim@gmail.com (D.V.); 4 IVF Athens Reproduction Center, 15123 Maroussi, Greece; diamathan16@gmail.com (D.A.); antoathan16@gmail.com (A.A.); diamathan17@gmail.com (A.A.); 5 King’s College Hospital NHS Foundation Trust, London SE5 9RS, UK; kyriakos.bananis@nhs.net; 6 Assisted Reproduction Unit, Fertility Institute, Paster 15, 11528 Athens, Greece

**Keywords:** oxidative stress, redox homeostasis, lipid peroxidation, granulosa cells, follicular fluid, polycystic ovary syndrome, diminished ovarian reserve, IVF outcomes, endometriosis, ovarian aging

## Abstract

Ferroptosis is a novel kind of regulated cell death that occurs when redox equilibrium is disrupted, leading to iron-dependent lipid peroxidation. Ferroptosis is defined by the buildup of deleterious lipid hydroperoxides, the inactivation of glutathione peroxidase 4 (GPX4), and mitochondrial shrinkage, setting it apart from apoptosis and necrosis. The relevance of this route to human reproduction remains unknown, despite its thorough investigation in neurodegeneration and cancer. Recent studies demonstrate that the ovarian follicular milieu is especially susceptible to ferroptosis owing to its high content of polyunsaturated fatty acids, iron-dependent metabolism, and the generation of reactive oxygen species. Dysregulation of ferroptosis may result in infertility by affecting granulosa cell survival, oocyte maturation, and embryonic competence. Ferroptotic activity correlates with oxidative stress indicators identified in clinical diseases including polycystic ovary syndrome, reduced ovarian reserve, and insufficient responsiveness to ovarian stimulation. Potential indicators include GPX4 expression, decreased glutathione levels, and the accumulation of lipid reactive oxygen species in granulosa cells and follicular fluid. Melatonin, which boosts antioxidant defences, and ferrostatin-1, a prototype inhibitor of ferroptosis that lowers lipid peroxidation, are two early candidates for treatment. For future evaluations, these agents should be used with standardised FF biomarker panels. Significantly, vitamin E, coenzyme Q10, and small-molecule ferroptosis inhibitors have shown efficacy in halting ferroptosis in experimental settings. These approaches have shown protective benefits in alternative systems and may signify viable treatment options for assisted reproduction. This narrative review encapsulates ferroptosis inside the ovarian follicle, its influence on oocyte quality, and the implications for in vitro fertilization results.

## 1. Introduction

Redox homeostasis, defined as the dynamic equilibrium between the continuous generation of reactive oxygen species (ROS) and reactive nitrogen species (RNS) and the function of enzymatic and non-enzymatic antioxidant defences, is crucial for cellular function [1]. Under physiological conditions, signalling cascades that regulate cell proliferation, differentiation, steroidogenesis, and responses to extracellular stimuli are fundamentally reliant on moderate concentrations of ROS, such as superoxide (O_2_^•−^), hydrogen peroxide (H_2_O_2_), and nitric oxide (NO^•^) [2]. For instance, nitric oxide regulates vascular tone and gametogenesis, while hydrogen peroxide functions as a second messenger in receptor tyrosine kinase signalling. Oxidative stress occurs when the production of ROS exceeds the capacity of antioxidant systems, such as glutathione peroxidases (GPXs), catalase, superoxide dismutases (SODs), and glutathione (GSH) [3]. This imbalance adversely affects macromolecules, destabilizes membranes, and compromises the integrity of the mitochondria and endoplasmic reticulum. Ferroptosis, a distinct and precisely controlled type of redox-dependent cell death, is characterised by a significant buildup of lipid peroxides in cellular membranes due to diminished antioxidant defences and elevated iron levels [4]. Ferroptosis is a unique mechanism of regulated cell death, mostly dependent on iron-mediated oxidative processes, unlike necrosis, which is marked by uncontrolled cell lysis, or apoptosis, which involves caspase activation and DNA breakage [5].

In 2012, Dixon and colleagues identified ferroptosis by noting that the RAS-selective deadly agent erastin, a tiny chemical, triggered a novel kind of cell death [6]. Erastin obstructed the cystine/glutamate antiporter system Xc^−^, including SLC7A11 and SLC3A2, by impeding cystine absorption, reducing intracellular cysteine levels, and constraining GSH production [7]. The Erastin administration induced an antioxidant deficiency, resulting in unregulated lipid peroxidation and iron-dependent oxidative cell death, since GPX4 function relies on GSH as a cofactor [8]. Cells subjected to erastin treatment had unique morphological characteristics, clearly distinguishing ferroptosis from apoptosis or necrosis: the nucleus retained structural integrity, although the mitochondria reduced in size, followed by heightened membrane density and the absence of cristae [9]. Ferrostatin-1, discovered in the same study, demonstrated that ferroptosis is a modifiable and pharmacologically targetable phenomenon, rather than an inevitable consequence of ROS accumulation, by acting as a radical-trapping antioxidant that can entirely prevent ferroptotic cell death [10]. This pioneering finding established the foundational principles of ferroptosis as a redox-driven, genetically encoded process of cell death, resulting in a decade of accelerated study into the implications of ferroptosis in health and illness [11].

The principal biochemical characteristic of ferroptosis is the buildup of lipid peroxides, especially in phospholipids rich in polyunsaturated fatty acids (PUFAs), subsequent to the deactivation of the cell’s protective enzymatic mechanisms [12]. The primary regulator of this process is GPX4, a selenoenzyme that converts membrane-bound lipid hydroperoxides (PUFA–PL–OOH) into their corresponding alcohols (PUFA–PL–OH) [13]. This inhibits lipid peroxidation chain reactions. GPX4 distinguishes itself from other members of the GPX family by its ability to directly decrease phospholipid hydroperoxides inside membranes. This illustrates the significance of inhibiting ferroptosis. Pharmacological inhibitors such as RSL3 or the genetic ablation of GPX4 lead to a rapid and detrimental accumulation of lipid peroxides [14]. Furthermore, when GSH levels are diminished due to cystine deprivation, suppression of system Xc^−^, or dysfunction in the γ-glutamyl cycle, cells are highly susceptible to ferroptosis. The presence of redox-active iron, which catalyses the Fenton reaction to generate hydroxyl radicals (^•^OH) from H_2_O_2_ and promote lipid peroxidation, further emphasises the vulnerability [15]. Ferroptosis incorporates many metabolic processes, including iron metabolism, glutathione dynamics, selenoprotein activity, and cystine transport.

The lipid content of cellular membranes significantly influences ferroptotic sensitivity. By enhancing membrane phospholipids with arachidonic acid (20:4) and adrenic acid (22:4), which are particularly prone to peroxidation within the ω-6 category [16]. Doll et al. (2017) assert that acyl-CoA synthetase long-chain family member 4 (ACSL4) functions as a molecular switch for PUFAs [17]. These fatty acids act as the principal substrates for iron-catalyzed lipid peroxidation after their esterification into phosphatidylethanolamines by lysophosphatidylcholine acyltransferase 3 (LPCAT3) [18]. Ferroptosis is not a uniform oxidative process; rather, it fundamentally depends on lipid remodelling enzymes that determine the pool of susceptible substrates, as shown by the notable resistance to ferroptosis in cells lacking ACSL4 [19]. In contrast, ferroptotic sensitivity is altered by the pharmacological suppression of ACSL4 or dietary modification of PUFA availability, indicating possible treatment approaches. Ferroptosis is a unique method of cell death triggered by the chemical composition of membrane lipids, rather than traditional caspase activation routes, owing to its strong connection with lipid metabolism [20].

GPX4-dependent defence plays a significant role in ferroptosis resistance; nevertheless, other antioxidant systems collaborate with it. Ferroptosis suppressor protein 1 (FSP1, sometimes referred to as AIFM2) was recognised as a robust glutathione-independent ferroptosis inhibitor in two separate investigations [4,21]. FSP1 is a NAD(P)H-dependent oxidoreductase that translocates to the plasma membrane via N-myristoylation and continuously produces reduced coenzyme Q10 (ubiquinol). CoQ10 is a lipophilic radical-trapping antioxidant that directly sequesters lipid peroxyl radicals (LOO^•^), therefore preventing their propagation along phospholipids containing polyunsaturated fatty acids (PUFAs) [22]. This pathway acts as an additional defence to GPX4, possibly allowing cells to withstand ferroptosis despite GSH depletion or GPX4 suppression, as long as FSP1-CoQ10 activity is maintained [23]. The discovery of FSP1 improved the understanding of ferroptosis control by indicating the existence of many, partly redundant protective mechanisms, including FSP1–CoQ10 at the plasma membrane and GPX4–GSH in the cytosol and mitochondria. Researchers are now investigating the use of pharmaceuticals to modify these pathways to either induce ferroptosis (as a cancer therapy) or inhibit it (to safeguard tissues from damage in degenerative illnesses) [24].

Ferroptosis is regulated by the interplay of GPX4 and FSP1, together with several transcriptional and organelle-specific networks. Mitochondria influence the dynamics of iron-sulfur clusters, alter the functionality of the electron transport chain, and generate ROS [25]. The endoplasmic reticulum regulates lipid desaturation and synthesises polyunsaturated fatty acid substrates that facilitate lipid peroxidation. Peroxisomes synthesise ether-linked phospholipids, which may enhance cellular sensitivity or provide protection, contingent upon the circumstances [26]. Ferritinophagy and other autophagic mechanisms enhance the labile iron pool and expedite ferroptosis by releasing free iron from ferritin stores. In varying cellular contexts, p53 may either enhance cellular susceptibility to ferroptosis or provide protection against it by modulating the activity of system Xc^−^ [27]. Conversely, the activation of Nrf2 at the transcriptional level initiates cytoprotective mechanisms, such as the upregulation of SLC7A11 and antioxidant enzymes. ATF4, HIF1α, and YAP/TAZ are additional transcription factors that integrate mechanotransduction, hypoxia signalling, and amino acid metabolism within the ferroptotic context [28]. Ferroptosis is a systemic phenomena that integrates iron biology, lipid remodelling, redox equilibrium, and organelle communication due to its multifaceted regulation [29].

The role of ferroptosis in human reproduction is largely unexamined, despite considerable studies in neurodegeneration, cancer biology, and ischemia–reperfusion damage. The ovarian follicle, owing to its distinct metabolic characteristics, is a milieu particularly prone to ferroptosis [30]. Granulosa and cumulus cells contain a significant amount of PUFAs and exhibit high activity in lipid metabolism, making them a substantial supply of substrates for lipid peroxidation [31]. Secondly, follicular fluid exhibits varying concentrations of ROS and contains redox-active iron, particularly during the peri-ovulatory period when the follicle ruptures, inducing inflammation-like processes [32]. Third, oocytes are susceptible to dysregulated lipid peroxidation because they rely on precise redox signalling for competence development and meiotic progression. Ferroptotic markers, including the downregulation of GPX4, the upregulation of ACSL4, and the accumulation of lipid peroxides, indicate oxidative stress and altered antioxidant capacity in pathological conditions such as PCOS, diminished ovarian reserve, premature ovarian insufficiency, and endometriosis [33]. The data indicate that ferroptosis may be a crucial but little investigated mechanism connecting oxidative stress to diminished egg quality, embryo viability, and in vitro fertilisation results.

Ferroptosis, when considered comprehensively, is a novel mechanism of cellular demise that alters our understanding of redox biology, lipid biochemistry, and iron metabolism. Lipid peroxidation was subsequently reinterpreted as an active biochemical mediator of cellular death rather than a passive consequence of oxidative stress [34]. Ferroptosis in a reproductive context may be examined in the ovarian follicle because of its unique metabolic and redox characteristics. Investigating ferroptosis in this setting may augment our comprehension of follicular physiology and promote the identification of innovative biomarkers and treatment approaches for ART [35]. A new approach to enhance oocyte quality and clinical outcomes in IVF may include targeting ferroptotic pathways by the modulation of iron availability, alteration of lipid composition, or reinforcement of antioxidant defences [36]. Ferroptosis might serve as a molecular cause of illness and an opportunity for application in reproductive medicine. In order to give a general idea of how ferroptosis works on a molecular level in relation to ovarian biology, Table 1 lists the main regulators, signalling pathways, and functional effects of ferroptosis.

Table 1 shows the main signalling pathways and ferroptosis regulators that are thought to be involved in ovarian dysfunction. The molecules on the list change iron metabolism, lipid peroxidation, or antioxidant defence, which affects the survival of granulosa cells, the maturation of oocytes, and fertility outcomes.

Table 1 illustrates the interaction of iron-handling mechanisms (NCOA4 ferritinophagy, HO-1 activity), lipid peroxidation inducers (ACSL4), and antioxidant defences (GPX4, FSP1, Nrf2/xCT) in regulating ferroptosis inside the ovarian follicle. PCOS, chemotherapy-induced POI, ovarian ageing, and endometriosis have all been shown to impair these regulators, correlating with diminished oocyte competence and infertility. Several pathways, including Nrf2 activation, ACSL4 inhibition, and GPX4 restoration, emerge as significant therapeutic candidates for enhancing ART outcomes.

## 2. Ovarian Environment and Fertility

### 2.1. PCOS—Human Evidence and Molecular Mechanisms

PCOS is the predominant endocrine condition among women of reproductive age and the primary aetiology of anovulatory infertility. Ovarian dysfunction has a complex aetiology including genetic, metabolic, and endocrine components [45]. Increasing data indicates that oxidative stress and redox imbalance are very relevant. Recent studies indicate that ferroptosis, an iron-dependent form of programmed cell death characterised by lipid peroxidation, significantly contributes to the pathogenesis of PCOS [46]. Li et al. (2024) presented the first direct data demonstrating that ovarian ferroptosis is elevated in women with PCOS and in rat models of PCOS generated by DHEA [40]. Granulosa cells from individuals with PCOS demonstrated increased levels of ferrous iron (Fe^2+^) and malondialdehyde (MDA), along with overexpression of nuclear receptor coactivator 4 (NCOA4) and concurrent downregulation of ferritin heavy chain 1 (FTH1) and GPX4 [47]. These alterations indicate the initiation of ferritinophagy. This mechanism selectively degrades ferritin, therefore releasing redox-active iron into the labile iron pool, facilitated by NCOA4 [48]. The resultant iron accumulation catalyses lipid peroxidation, hence facilitating ferroptosis and diminishing the viability of granulosa cells. The prevention of ferroptosis by ferrostatin-1 improved many symptoms of PCOS in rats, such as anovulation, hyperandrogenism, glucose intolerance, and delayed oocyte maturation, while also restoring follicular shape [49]. The causal relationship between androgen excess, ferroptotic cell death, and reproductive failure was strengthened when ferroptosis was pharmacologically stimulated with RSL3, worsening PCOS symptoms.

Transcriptomic investigations, extending beyond experimental models, have validated the involvement of ferroptosis in PCOS. Huang et al. (2024) discovered 14 differentially expressed ferroptosis-related genes (FRGs) using bioinformatics analysis of granulosa cell datasets (GSE155489, GSE168404) [50]. These were more prevalent in biological processes related to antioxidant defences, the integrity of the mitochondrial outer membrane, and the metabolism of ROS. Upon validation, eight major FRGs (ATF3, BNIP3, DDIT4, LPIN1, NOS2, NQO1, SLC2A1, SLC2A6) shown substantial diagnostic effectiveness in differentiating individuals with PCOS from those without [51]. Their functional association with oocyte quality metrics such as retrieval rate, MII maturation rate, fertilisation efficiency, and generation of high-quality embryos underscores the significance of these genes for translation. NOS2 enhances nitric oxide production, exacerbating nitrosative stress [52]. LPIN1 regulates lipid catabolism and their propensity for oxidation. BNIP3 and DDIT4 are stress-responsive genes that connect hypoxia and mTOR signalling to mitochondrial metabolism [53]. The quintessential Nrf2 target, NQO1, exhibits compensatory antioxidant responses. This transcriptional profile linked to ferroptosis highlights that PCOS granulosa cells have a chronic redox imbalance, marked by the overexpression of pro-oxidant pathways and inadequate antioxidant defences, thus compromising oocyte support [54]. Furthermore, network analysis identified HMGA1 and JUN as transcriptional regulators. Computational drug repurposing indicated that dicoumarol and flavin adenine dinucleotide may influence ferroptosis pathways, potentially resulting in novel therapeutic alternatives in the future [55].

Non-coding RNAs, particularly circular RNAs (circRNAs), contribute to the regulation of ferroptosis in granulosa cells of PCOS. Zhang et al. (2021) discovered that circRHBG levels were significantly elevated in granulosa cells of women with PCOS [56]. Functional experiments demonstrate that circRHBG enhances granulosa cell proliferation by altering ferroptosis resistance. CircRHBG functions as a competitive endogenous RNA (ceRNA) that alleviates the inhibition of SLC7A11, the light chain component of system Xc^−^, by sequestering miR-515-5p [57]. This inhibits ferroptosis by enhancing cystine absorption, replenishing GSH levels, and maintaining elevated GPX4 activity. Inhibition of circRHBG resulted in a reduction in viable granulosa cells and an increase in lipid peroxidation [58]. This indicates that the circRHBG/miR-515-5p/SLC7A11 axis functions as a preventive mechanism against ferroptosis in the ovary affected by PCOS. This regulatory circuit illustrates how post-transcriptional regulation influences ferroptotic sensitivity by modulating cystine availability, GSH metabolism, and GPX4 functionality [59]. Moreover, it indicates that PCOS granulosa cells may use circRNA-mediated mechanisms to acclimatise to hyperandrogenic and chronic oxidative stresses, albeit leading to follicular stoppage and abnormal proliferation.

Iron metabolism serves as a significant connection between ferroptosis and the pathogenesis of PCOS. Zhang et al. (2021) discovered that granulosa-like KGN cells under iron-rich conditions have an increased expression of the transferrin receptor (TFRC), resulting in excessive uptake of Fe^2+^ [56]. The iron excess activated NADPH oxidase 1 (NOX1), resulting in increased ROS and mitochondrial damage. TFRC activation initiated mitophagy via PTEN-induced kinase 1 (PINK1), which further regulates iron turnover and redox state [60]. TFRC activation also elevated ACSL4, which was very essential. This altered the lipid composition of membranes to promote substrates susceptible to peroxidation. The interplay of NOX1-derived reactive oxygen species, PINK1-dependent mitophagy, and ACSL4-mediated lipid remodeling established a pro-ferroptotic milieu in both viable and non-viable cells [61]. This environment was marked by GPX4 degradation, accumulation of lipid peroxides, and disrupted folliculogenesis. Knockdown of TFRC or inhibition of downstream effectors reduced ferroptosis and partly restored granulosa cell survival, suggesting that iron absorption and redox interactions may represent viable therapeutic targets [62]. The findings substantiate the hypothesis that PCOS is a condition predisposed to ferroptosis, characterized by oxidative stress, lipid peroxidation, and impaired iron metabolism, which collectively impede follicle development, rather than being solely an endocrine disorder defined by hyperandrogenism and insulin resistance.

Ferroptosis is believed to contribute to the pathogenesis of PCOS through several interrelated mechanisms: (i) androgen-induced ferritinophagy releases iron and diminishes GPX4 levels, resulting in lipid peroxidation and the apoptosis of granulosa cells; (ii) transcriptomic reprogramming of granulosa cells enhances ferroptosis-related gene signatures associated with diminished oocyte quality; (iii) circRNA–miRNA regulatory networks reduce ferroptosis sensitivity by modulating cystine uptake and antioxidant defences; and (iv) iron overload due to TFRC, in conjunction with NOX1 activation and ACSL4-mediated lipid remodelling, accelerates ferroptotic damage [63]. These molecular anomalies are indicative of PCOS and directly influence oocyte quality, fertilization success, and embryo quality during IVF. Therapeutic targeting of ferroptotic pathways, via radical-trapping antioxidants, iron chelation, or regulation of system Xc^−^ activity, may provide novel avenues to restore reproductive function in afflicted women. Pharmacological suppression of ferroptosis in animal models exacerbates PCOS characteristics.

The degree of evidence and implications for ART in PCOS indicate that ferroptosis has moderate overall support, with credible associations to reduced fertilisation and embryo quality. Nevertheless, consistent but mechanistically varied human follicular fluid redox/oxidation-reduction potential signals are necessary for more robust conclusions. Standardised panels that consider BMI are necessary.

### 2.2. POI and Chemotherapy-Induced Ferroptosis in Ovarian Granulosa Cells

Chemotherapy-induced ovarian toxicity is one of the main reasons why young cancer patients have POI. Stromal fibrosis and oocyte apoptosis were the primary etiological factors of this damage in the past. Recent studies demonstrate that GC ferroptosis is a critical mechanism responsible for chemotherapy-induced POI [64]. CTX and cisplatin (Cis) not only damage DNA but also disrupt redox homeostasis, leading to iron overload, lipid peroxidation, and mitochondrial dysfunction. Chemotherapy in GCs diminishes antioxidant defenses such as GPX4 and GSH, while augmenting the labile iron pool and facilitating the generation of ROS across various compartments, including cytosolic, lipid, and mitochondrial ROS [65]. These conditions work together with changes in lipid metabolism, especially the upregulation of ACSL4, which adds polyunsaturated fatty acids that are prone to peroxidation to membranes. The outcome is distinctive ferroptotic morphology (condensed mitochondria with ruptured cristae), abnormal follicular development, and, ultimately, ovarian fibrosis and endocrine failure [66]. These findings suggest that chemotherapy-induced POI is a ferroptosis-driven phenomenon at the nexus of oxidative stress, lipid remodeling, and iron metabolism, rather than a mere outcome of apoptosis.

Chen et al. (2024) demonstrated that heme oxygenase-1 (HO-1) and mitochondrial dysfunction contribute to the direct induction of ferroptosis in GCs by CTX. RNA-seq analysis of COV434 and KGN cells treated with CTX revealed enrichment in the ferroptosis pathway [29]. This was shown by the fact that GPX4 was turned down and HO-1 was turned up. Functionally, CTX exposure led to significant ultrastructural mitochondrial abnormalities, a reduction in mitochondrial membrane potential, elevated lipid peroxidation, iron accumulation, and an overload of ROS in the mitochondria [67]. HO-1 overexpression broke down heme to free iron, which increased the production of ROS through Fenton chemistry and overpowered defences that depend on GPX4. Pharmacological inhibition of HO-1 significantly diminished ferroptosis and mitigated GPX4 depletion, underscoring HO-1 as a primary factor in CTX-induced GC mortality [68]. This axis connects lipid peroxidation and mitochondrial dysfunction to heme catabolism and iron release. It suggests that HO-1 could be a good target for protecting women’s ovaries when they are taking alkylating agents.

Cisplatin, another chemotherapy drug that is often used, has also been shown to cause ferroptosis in ovarian tissue. Wang et al. (2023) said that cis treatment led to iron buildup in the ovaries, ferroptotic GC death, and tissue damage that ferrostatin-1 could fix [59]. At the molecular level, Cis significantly enhanced the activity of ACSL4, an enzyme that transforms ω-6 PUFAs into membrane phospholipids, thereby increasing cellular susceptibility to peroxidation [69]. It was found that the transcription factor SP1 directly binds to the promoter of ACSL4, which makes it overexpress when Cis stress is present. Consequently, the transcriptional activation of ACSL4 mediated by SP1 was a critical switch for chemotherapy-induced ferroptosis [70]. The pharmacological ACSL4 inhibitor rosiglitazone treatment diminished ovarian damage and enhanced GC survival in vivo. This study identified transcriptional regulation (SP1–ACSL4 axis) as a novel therapeutic vulnerability in chemotherapy-associated ovarian injury, while also establishing ACSL4 as a crucial mediator of Cis-induced ferroptosis.

Du et al. (2023) established a further connection between ferroptosis and ovarian tissue fibrosis, a hallmark of POI [71]. Using a rat model, they showed that Cis exposure caused fibrotic remodelling of the ovarian stroma, hormonal insufficiency, and disordered folliculogenesis. Ferroptosis was shown by a lot of iron buildup in ovarian tissue, higher MDA levels, and mitochondria getting smaller. Molecular profiling confirmed the ferroptotic phenotype by showing that SLC7A11 and GPX4 were turned down and ACSL4 and ALOX15 (a lipoxygenase enzyme that increases lipid peroxidation) were turned up. Interestingly, vitamin E, a traditional lipophilic antioxidant and radical-trapping agent, reversed many of these effects, improving fertility outcomes, reducing fibrosis, and restoring ovarian function in treated animals. This study emphasizes ferroptosis as a contributor to fibrotic remodelling alongside GC loss, suggesting that ferroptosis inhibitors may alleviate the extracellular and cellular consequences of chemotherapy-induced POI [71].

Targeting ferroptosis pathways with stem cell-based interventions is a particularly compelling therapeutic strategy. Zhou et al. (2024) showed that exosomes from human umbilical cord mesenchymal stem cells (hUMSC-Exos) protect ovarian function in mice with CTX-induced POI by blocking ferroptosis through the Nrf2/GPX4 signalling pathway [35]. Exosome therapy brought back the levels of Nrf2, xCT (SLC7A11), and GPX4 in GCs while lowering ROS, iron overload, and lipid peroxidation. Blocking Nrf2 with ML385 got rid of these protective effects, which shows that exosomal cargo boosts antioxidant defences by activating Nrf2. In a similar vein, Dai et al. (2024) found that hUC-MSCs lessen CTX-induced POI by stopping ferritinophagy-mediated ferroptosis, which keeps ferritin stores from letting out too much iron [22]. Pan et al. (2024) provide complementary evidence that endometrial stem cells inhibit Cis-induced ferroptosis in GCs via Nrf2-dependent mechanisms, underscoring a common protective pathway among various stem cell types [72]. All of these studies indicate that stem cell-derived exosomes and cellular therapies are promising strategies for safeguarding folliculogenesis, maintaining fertility in chemotherapy patients, and recalibrating ferroptotic sensitivity in granulosa cells.

These findings delineate a convergent model in which chemotherapy agents induce ferroptosis in granulosa cells through distinct yet intersecting pathways: Cisplatin via SP1-mediated ACSL4 overexpression and ALOX15 activation, Cyclophosphamide through HO-1–ROS–mitochondrial dysfunction, and both through lipid peroxidation, iron overload, and GPX4 depletion [73]. This ferroptotic damage not only reduces GC viability but also induces ovarian fibrosis, hormonal imbalance, and ultimately the POI phenotype. Several lines of evidence strongly suggest that ferroptosis inhibition can mitigate ovarian damage [74]. This can be accomplished through Nrf2-targeted exosome therapies, small-molecule inhibitors such as ferrostatin-1, ACSL4 blockade via rosiglitazone, or radical-trapping antioxidants like vitamin E. These advancements provide a rational basis for the development of fertility-preserving strategies for young cancer survivors and identify ferroptosis as a mechanistic connection between chemotherapy and reproductive toxicity.

The calibre of evidence and its ramifications for ART (area of focus): Ferroptosis plays a significant role in preclinical studies, as demonstrated by inhibitor rescue and reliance on GPX4, and exhibits moderate involvement in clinical settings, correlating with follicle loss and diminished ovarian reserve; therefore, strategies that modulate ferroptosis merit translational evaluation regarding fertility preservation.

### 2.3. Ferroptosis, Ovarian Senescence, and Reduced Ovarian Reserve

Ovarian aging is characterized by a steady drop in both the amount and quality of oocytes, eventually resulting in decreased reproductive potential and DOR [75]. Recent transcriptomic and functional investigations suggest that ferroptosis plays a crucial role in the age-related loss of oocytes and granulosa cells, despite apoptosis and mitochondrial malfunction being historically linked to this decrease [76]. This notion is based on the discovery that oxidative damage, iron buildup, and lipid peroxides—molecular indicators of ferroptosis exist in aging ovaries. In aged oocytes, mitochondria produce less ATP, have compromised cristae integrity, and generate increased ROS, all of which heighten the susceptibility of cells to lipid peroxidation [47]. The reduced expression of ferroptosis defense genes, including GPX4 and SLC7A11, is significantly associated with these structural and metabolic changes, shifting the balance towards ferroptotic cell death. With advancing age, the ovarian follicular milieu becomes predisposed to ferroptosis due to the insufficient protective capacity of granulosa cells against oxidative damage, coupled with the accumulation of lipids in oocytes that are susceptible to peroxidation [77].

Jia et al. (2023) presented actual human data by examining granulosa cells from women of AMA with either normal ovarian response (NOR) or diminished ovarian reserve (DOR). Over 6000 genes were identified as differently expressed, including ferroptosis, mitochondrial pathways, and oxidation-reduction activities [37]. Granulosa cells from women with AMA-DOR had a transcriptional profile suggestive of heightened susceptibility to lipid peroxidation, altered metal ion binding, and impaired energy metabolism. KEGG pathway study indicated that ferroptosis and oxidative phosphorylation have dual roles as both causal and consequence factors in mitochondrial dysfunction [37]. The results indicate that the activation of ferroptosis may clarify the varied clinical outcomes seen in AMA patients. Individuals who maintain normal reserves may have an increased ability to suppress ferroptotic pathways, hence preserving oocyte quality and improving IVF success rates. This research demonstrates that ferroptosis is a biological component affecting reproductive heterogeneity in older women.

Lin et al. (2023) used patient biopsies, single-cell RNA sequencing, and spatial transcriptomics to clarify gene expression patterns associated with ferroptosis in ovarian senescence [73]. Their multi-omic research demonstrated the overexpression of TFRC (transferrin receptor), NCOA4 (ferritinophagy mediator), and SLC3A2 (system Xc component), which correlates with enhanced iron absorption, ferritin breakdown, and dysregulation of cystine transport [78]. Concurrently, GPX4 was downregulated, which directly impeded the detoxification of lipid peroxides. These alterations together render the redox state more conducive to lipid peroxidation and iron accumulation. Mechanistically, aged ovaries demonstrate a reorganisation of mitochondrial metabolism, marked by increased ROS leakage from the electron transport chain (ETC) and reduced efficiency of the TCA cycle [79]. This metabolic stiffness heightens susceptibility to ferroptosis, therefore influencing lipid peroxidation and mitochondrial dysfunction. Significantly, these molecular alterations were restored with dietary treatment with DHEA, ubiquinol CoQ10, and Cleo-20 T3, therefore improving oocyte competence and restoring GPX4 expression [80].

Genetic mutations, in conjunction with age and metabolism, may contribute to ovarian insufficiency associated with ferroptosis. Wang et al. (2022) discovered that a Chinese family with hereditary primary ovarian insufficiency has mutations in Basonuclin 1 (BNC1) [38]. BNC1 deficiency resulted in premature follicle activation, excessive atresia, and complications in lipid metabolism. The absence of BNC1 triggered the NF2-YAP pathway, which regulates Hippo signaling, resulting in oocyte death by ferroptosis [38]. Pharmacological suppression of YAP or ferroptosis notably improved ovarian function in Bnc1-mutant animals, restoring follicle integrity and hormone output. This work illustrates that ferroptosis may result from genetic abnormalities that impair lipid homeostasis and redox control, and it is also linked to ageing. The association between ferroptosis and transcriptional regulators (BNC1, YAP) further illustrates the interplay between redox metabolism and nuclear signaling pathways in regulating ovarian reserve [38].

Recent findings suggest that ferroptosis in granulosa cells may have a role in the development of ovarian stromal fibrosis, a notable feature of ovarian aging, as well as the decline in oocyte quality [81]. Ferroptotic granulosa cells emit lipid peroxidation byproducts, including 4-hydroxynonenal (4-HNE) and DAMPs, which stimulate fibroblasts and facilitate extracellular matrix remodeling. The fibrotic microenvironment establishes a detrimental cycle that accelerates follicle loss by obstructing blood supply to the follicles and elevating oxidative stress [82]. Ferroptosis and ovarian fibrosis have been associated in animal models of chemotherapy-induced ovarian insufficiency, indicating that analogous processes may contribute to normal ovarian aging. In this scenario, ferroptosis serves as a paracrine inducer of tissue remodeling and degeneration while functioning as a cell-autonomous death pathway [71].

Ferroptosis plays a crucial role in DOR and ovarian aging, prompting the investigation of various therapeutic modalities. Vitamin E and ferrostatin-1 exemplify radical-trapping antioxidants that function in preclinical ovarian models and inhibit lipid peroxidation [29]. Nutritional therapies using CoQ10, DHEA, and other mitochondrial cofactors enhance GPX4 activity and restore mitochondrial efficiency. Age-related diminished ovarian reserve may be improved by stem cell-derived exosomes, an innovative bioactive treatment now being studied in primary ovarian insufficiency, which elevates Nrf2 and GPX4 expression while mitigating iron overload [83]. Furthermore, chelators or NCOA4 inhibition, which disrupt iron homeostasis, may obstruct ferritinophagy-mediated ferroptosis in granulosa cells. Collectively, these tactics indicate that ferroptosis may serve as a therapeutic approach to address reproductive aging, perhaps enabling older women to achieve prolonged ovarian function and improved IVF outcomes [84].

The strength of evidence and implications for ART (Aging/DOR) indicate that mechanistic plausibility, including iron accumulation, PUFA peroxidation, and mitochondrial ROS, is more significant than conflicting findings from human FF studies; targeted antioxidant and lipid-peroxidation management may enhance oocyte competence, pending future validation.

The collected data suggests ferroptosis as a unified process in ovarian aging, connecting transcriptional dysregulation, mitochondrial dysfunction, iron metabolism, and granulosa cell failure [63]. Transcriptomic patterns associated with ferroptosis differentiate older women with ovarian reserve from those with DOR, indicating that the suppression of ferroptosis may be fundamental to preserving fertility. In preliminary clinical and experimental settings, dietary and pharmacological therapies targeting ferroptosis pathways have shown encouraging results, establishing a translational foundation for methods to maintain fertility [63]. Ferroptosis serves as the ultimate executioner in hereditary primary ovarian insufficiency disorders, as shown by genetic models like BNC1 loss, underscoring its significant importance beyond age-related decline. Future clinical research should focus on identifying ferroptosis biomarkers in granulosa cells and follicular fluid, validating ferroptotic signals in IVF populations, and assessing particular inhibitors in controlled trials. Therefore, elucidating ferroptosis in connection with the ovaries may augment our knowledge of reproductive aging and enable tailored interventions to combat infertility in women with reduced ovarian reserve.

Nearly fifty percent of women with infertility have endometriosis, and ovarian endometriomas further impede follicular development. A notable biochemical characteristic of ovarian endometriosis is the excessive accumulation of iron in the follicular fluid, resulting from the recurrent breakdown of red blood cells inside endometriotic cysts [41]. This extended exposure to iron fosters an optimal milieu for oxidative stress and ferroptosis. Ni et al. (2022) shown that FF from women with EMFF directly triggers ferroptotic cell death in GCs in both in vitro and in vivo settings [41]. EMFF enhanced the release of iron from ferritin and promoted Fenton chemistry by increasing the levels of Fe^2+^ and malondialdehyde (MDA, a sign of lipid peroxidation), while also triggering ferritinophagy dependent on NCOA4. When GPX4 levels were markedly reduced, the primary antioxidant defence against lipid peroxidation was compromised. Transmission electron microscopy validated the ultrastructural characteristics of ferroptosis, including broken cristae and reduced mitochondria with electron-dense membranes [85]. Ferroptosis in granulosa cells compromised the oocyte microenvironment, resulting in suboptimal maturation of both the cytoplasm and nucleus. Ferroptotic GCs generated exosomes with pro-oxidant cargo, exacerbating follicular damage by disseminating the issue to adjacent cells [86]. Women with ovarian endometriosis have inferior fertilisation results compared to those with peritoneal endometriosis. This results from the dual impact of direct germ cell loss and indirect exosome-mediated oocyte dysfunction. In preclinical models, the therapeutic treatment of vitamin E, an antioxidant that sequesters free radicals, and deferoxamine, an iron chelator, markedly decreased GC ferroptosis, restored oocyte viability, and enhanced the quality of IVF embryos [87]. These findings demonstrate the translational importance of focussing on ferroptotic pathways for fertility preservation and establish ferroptosis as the molecular link between iron-overloaded follicular fluid and endometriosis-related infertility.

Granulosa cells have a pronounced susceptibility to ferroptosis, whereas ectopic ESCs in ovarian endometriomas demonstrate a more intricate, dual response. Dong et al. (2023) indicated that embryonic stem cells exposed to excess iron in ectopic lesions experienced autophagy-dependent ferroptosis, marked by altered mitochondrial morphology, heightened lipid peroxidation, and decreased viability [88]. Autophagy, often a protective cellular process, functioned as a pro-ferroptotic mechanism in this instance by degrading ferritin, hence augmenting the labile iron pool and accelerating ROS-mediated lipid peroxidation [89]. The reliance was confirmed in vivo, since autophagy suppression reduced ferroptotic indicators in EM lesions. Ironically, endometriotic lesions exhibit resistance to full ferroptotic eradication. Transcriptomic investigations indicate that ATF4 (activating transcription factor 4) is overexpressed in ectopic tissues [90]. This enhances the functionality of the cystine/glutamate antiporter (system Xc^−^, composed of SLC7A11 and SLC3A2). This mechanism neutralizes lipid peroxides and maintains GSH production, intracellular cystine replenishment, and GPX4 function. Ectopic lesions exist in a paradoxical state, simultaneously protected by ATF4–xCT-mediated antioxidant reinforcement, which promotes their survival and proliferation in a hostile microenvironment, while also being exposed to high-iron, high-ROS conditions that trigger ferroptotic signalling [23]. This molecular adaptation illustrates the distinct nature of ferroptosis in EM: granulosa cells undergo ferroptosis, while stromal cells establish protective networks for survival. Thus, targeting ATF4 or xCT may elucidate the susceptibility of stromal cells to ferroptosis, providing a method to selectively eradicate endometriotic tissue while preserving ovarian reserve.

A significant aspect of endometriosis is the role of ferroptosis in inducing fibrosis, which is a crucial component of chronic endometriotic lesions. Zhang et al. (2022) assert that iron concentrations in ectopic ovarian stromal tissue significantly exceed those in eutopic endometrium [39]. This induces ferroptosis in local MSCs, leading to their demise. Alongside the elevation of ferroptotic markers and lipid peroxidation, the in vitro exposure of ESCs to FAC resulted in a significant phenotypic alteration: a reduction in MSCs and an increase in fibroblasts and α-SMA-positive myofibroblasts [91]. This change facilitates the buildup of extracellular matrix, ultimately solidifying the ovarian milieu and impairing follicular activity. Inducing ferroptosis exacerbated fibrosis in vivo; however, inhibiting ferroptosis with ferrostatin-1 or chelating iron with deferoxamine ameliorated the fibrotic alterations. Ferroptotic byproducts, including oxidized phospholipids and 4-hydroxynonenal (4-HNE), presumably function as DAMPs, activating fibroblasts and sustaining inflammation and fibrosis. The paracrine action of ferroptosis significantly modifies the stromal milieu, making it unfavorable for oocyte growth and ovulation, while concurrently killing individual cells. Ferroptosis links excessive iron to the prolonged depletion of ovarian reserve seen in endometrioma patients by acting as a cellular execution mechanism and a catalyst for enduring tissue remodeling [92].

Ferroptosis is recognised as a dual-faceted mechanism in endometriosis, substantiated by increasing data [90]. Ferroptosis adversely affects oocytes, embryos, and granulosa cells, directly resulting in sterility. Elevated iron concentrations in follicular fluid, peritoneal fluid, and ectopic cysts diminish embryo quality, accelerate lipid peroxidation, and impede communication between granulosa cells and oocytes [90]. Conversely, ferroptosis may inhibit tumour proliferation by preventing the development of ectopic lesions in iron-rich environments. As previously mentioned, stromal cells trigger the ATF4–xCT–GPX4 defence mechanism to prevent ferroptotic death, hence allowing lesions to persist. This contradiction presents a therapeutic dilemma: the sensitization of ferroptosis to reverse endometriotic lesions and the suppression of ferroptosis to preserve folliculogenesis and IVF results must be carefully managed [4]. The dichotomy exemplifies oncological concepts in which ferroptosis functions as both a therapeutic potential in resistant tumours and a detriment in normal tissue. To address this conundrum in reproductive medicine, it is crucial to selectively target cell types, which involves sensitising endometriotic stromal cells to ferroptosis while maintaining the integrity of granulosa cells and oocytes. This therapy may accomplish two therapeutic objectives concurrently by enhancing fertility and slowing disease progression.

The increasing significance of ferroptosis in EM-related infertility underscores the need for translational techniques. Antioxidants such as vitamin E and liproxstatin-1, iron chelators such as deferoxamine, and NCOA4 inhibitors that decrease ferritinophagy may safeguard granulosa cells and oocytes against ferroptosis. Targeting ATF4–xCT signaling or using pharmacological inducers of ferroptosis may increase the susceptibility of ectopic stromal cells, hence diminishing lesion size and fibrotic remodeling [93]. The prognosis of IVF in women with endometriosis may be elucidated by minimally invasive biomarkers of follicular ferroptosis obtained via profiling exosomes generated by ferroptotic granulosa cells. To preserve reproductive capability and inhibit lesion development, advanced methods may combine systemic antioxidants with localized ferroptosis modulators applied to endometriotic cysts. Ferroptosis unifies several characteristics of endometriosis into a comprehensive molecular framework, including iron excess, oxidative stress, chronic inflammation, fibrosis, and infertility. This integrative approach not only improves our comprehension of EM pathophysiology but also creates new treatment opportunities at the intersection of targeted cell death control, redox medicine, and reproductive biology. Table 2 delineates significant oxidative stress indicators, their variations, and associated reproductive outcomes.

Table 2: A compilation of oxidative stress markers in follicular fluid and how they relate to IVF results. Biomarkers like GSH, MDA, ORP, and 8-OHdG are always linked to how well fertilisation works, how good the embryos are, and how the pregnancy is going. Other biomarkers, including TAC, do not consistently exhibit analogous associations. These findings collectively underscore the translational potential of redox markers in forecasting ART efficacy.

## 3. Follicular-Fluid Redox and IVF Outcomes: Bridging Mechanistic Insights to Clinical Endpoints

The oocyte undergoes maturation and prepares for fertilization inside the dynamic milieu known as follicular fluid. The metabolites, growth factors, hormones, and redox regulators originate from the granulosa and theca cells, the circulating plasma, and the ovarian milieu [101]. The intricate equilibrium between ROS and antioxidant defenses facilitates follicle growth, meiotic advancement, and oocyte competence. At the physiological level, ROS function as signaling molecules for ovulation, fertilization, and cumulus expansion [36]. Oxidative stress, resulting from an excess of ROS or insufficient antioxidant defenses, destroys lipids, proteins, and DNA in the oocyte and adjacent cells. Human clinical investigations indicate that oxidative imbalance in follicular fluid is consistently associated with reduced fertilization rates, diminished embryo quality, and decreased implantation potential [102]. Nishihara et al. found that 8-hydroxy-2′-deoxyguanosine (8-OHdG), a marker of DNA oxidation, was elevated in patients with both insufficient fertilisation and blastocyst development, while total GSH, a key antioxidant, was significantly diminished in women with suboptimal ICSI fertilisation rates [94]. The results indicate that an oxidatively stressed follicular fluid environment may have lasting impacts on the integrity of the embryonic genome, as well as impede the first sperm-oocyte union. It was evident that GSH levels were reduced in individuals with endometriosis. This indicates that the condition diminishes the body’s capacity to combat free radicals, resulting in iron accumulation and chronic inflammation. These data suggest that FF redox equilibrium is a crucial factor influencing ART outcomes and a potential source of prognostic biomarkers.

Global measurements such as ORP consider the comprehensive balance of all oxidant and reductant species present, while individual markers provide more detailed insights into operational mechanisms. ORP may facilitate clinical translation by providing a singular, quantitative representation of the redox status [103].

Researchers have investigated the efficacy of several FF molecules in predicting IVF results. MDA, a consequence of lipid peroxidation, is among the most extensively researched substances. Increased FF MDA compromises membrane integrity and cellular signalling by indicating excessive ROS-induced peroxidation of polyunsaturated fatty acids in granulosa cell and oocyte membranes [104]. Yalçınkaya et al. demonstrated that women who conceived had significantly elevated levels of MDA, as shown by an ROC AUC of 0.74 for predicting conception [96]. This unexpected finding indicates that, in contrast to pathological oxidative stress, a certain level of lipid peroxidation may signify physiological ROS signaling essential for ovulation and fertilization. Similarly, research indicates that the redox-active signalling molecule NO affects sperm-oocyte interactions, meiotic resumption, and vascularization. Pregnant women within the same cohort had reduced levels of FF NO, suggesting that excessive NO may induce nitrosative stress or disrupt cellular signalling mechanisms. Provided that concentrations remain within a regulated range, more study on NO_2_^−^/NO_3_^−^ corroborates its beneficial effects on fertilisation and embryo cleavage [105]. Finally, DNA oxidative damage, shown by 8-OHdG levels, has consistently been associated with diminished fertilisation and blastocyst quality. These data suggest that interpreting FF indicators requires complexity; the strength, timing, and molecular context of oxidative processes determine whether redox signals are advantageous or harmful [102]. An integrated methodology that incorporates antioxidant capacity (GSH, TAC), oxidative damage indicators (MDA, 8-OHdG), and signalling molecules (NO) offers the most lucid understanding of FF biology.

Underlying gynecological problems substantially influence follicular fluid redox patterns, often resulting in disease-associated subfertility. Studies on controlled ovarian stimulation in endometriosis reveal markedly increased follicular fluid malondialdehyde levels compared to tubal-factor controls, indicating iron-mediated reactive oxygen species buildup due to bleeding in endometriotic cysts [106]. Patients with endometriosis under 33 years of age had increased oxidative indicators while achieving relatively greater conception rates. This signifies that the inherent resistance of oocytes declines with age, exacerbating the adverse impacts of oxidative stress [107]. Cross-sectional research and comprehensive reviews indicate that women with PCOS have heightened oxidative stress, chronic inflammation, modified growth factor signalling, and disordered metabolic hormones. These fingerprints align with mechanistic investigations into inflammatory stress and ferroptosis in PCOS granulosa cells, where an impaired redox balance obstructs embryonic competence and oocyte–granulosa cell communication [108]. The redox pathophysiology of the two illnesses exhibits both distinctions and similarities. PCOS is characterized by chronic low-grade inflammation and an abundance of metabolic ROS, while endometriosis mostly arises from iron accumulation and conditions favorable to ferroptosis. The identification of these disease-specific signals enhances our mechanistic understanding and enables personalized biomarker-driven prognostics in ART [109].

If the redox condition of follicular fluid dictates IVF success, modifying redox pathways is a logical therapy approach. While non-pregnant women show signs of antioxidant depletion, observational studies consistently demonstrate that women who conceive via IVF have increased levels of follicular fluid total antioxidant capacity, glutathione, catalase, and superoxide dismutase activity [110]. The results of dietary and pharmaceutical therapies designed to restore redox equilibrium have been ambiguous. Supplementation with vitamins C and E, CoQ10, and melatonin has been shown to improve follicular fluid antioxidant profiles, oocyte maturation, and embryo development; nevertheless, randomised studies concerning pregnancy and live birth outcomes have produced contradictory findings. The variability is likely attributable to disparities in ovarian reserve, disease phenotypes, and baseline redox state [111]. Targeted strategies exhibit improved effectiveness: anti-inflammatory antioxidants for PCOS, mitochondrial cofactors (CoQ10, DHEA) for advanced maternal age and DOR, and iron chelators for endometriosis. Innovative approaches, including stem-cell-derived exosomes that enhance Nrf2 and GPX4 activity in granulosa cells, may expand therapeutic alternatives [112]. To determine which patients are most likely to benefit from therapies, it is crucial for clinical translation to classify individuals based on baseline FF redox biomarkers using ORP, TAC, and oxidative damage indicators. Antioxidant therapy in IVF may go from empirical supplements to precision redox medicine.

## 4. Translational Pathways and Therapeutic Targets in Reproductive Ferroptosis

Nrf2 serves as the primary transcriptional regulator of cellular antioxidant defence and the inhibition of ferroptosis. It serves as the molecular switch that determines whether a cell adapts to oxidative stress or succumbs to lipid peroxidation. KEAP1 interacts with inactive Nrf2 and facilitates its degradation via ubiquitination [113]. Oxidative or electrophilic stress modifies critical cysteine residues on KEAP1, facilitating the translocation of Nrf2 into the nucleus, where it interacts with AREs in the promoters of cytoprotective genes [114]. The primary enzyme responsible for the degradation of lipid hydroperoxides is GPX4, whereas SLC7A11 (xCT) functions as the cystine/glutamate antiporter essential for GSH synthesis. The conventional method to safeguard against ferroptosis involves the Nrf2-xCT-GSH-GPX4 pathway [85]. Toxins, environmental contaminants, or ageing may disrupt this process in ovarian granulosa cells, diminishing antioxidant reserves and making the follicular milieu more vulnerable to ferroptosis. Zhang et al. (2024) explicitly shown that exposure to PM2.5 triggers ferroptosis in granulosa cells by diminishing GSH levels, downregulating GPX4, and increasing MDA synthesis coupled with Fe^2+^ buildup [85]. Nrf2 mutant mice demonstrated accelerated ovarian failure, highlighting its critical function in ovarian resilience. Restoration of Nrf2 activity, either pharmacological activators such as melatonin or genetic overexpression, completely restored GPX4/xCT expression, decreased lipid peroxidation, and maintained follicular integrity [115]. Melatonin prolonged transcriptional activity by promoting Nrf2 nuclear translocation and obstructing KEAP1-mediated ubiquitination. The results suggest that Nrf2 is the primary regulator of ovarian ferroptosis, and altering its activity may affect the transition of granulosa cells to ferroptotic collapse or the preservation of redox homeostasis. Nrf2 activators are intriguing possibilities for protecting women’s fertility against environmental contaminants or gonadotoxic treatments [116]. They may also serve as the foundation for adjuvant regimens in IVF operations for individuals experiencing infertility linked to oxidative stress.

Melatonin, traditionally acknowledged for its neuroendocrine and circadian functions, has lately been established as a powerful antioxidant that selectively targets mitochondria and modulates apoptosis [117]. Its amphiphilic nature facilitates effortless membrane traversal and accumulation inside mitochondria. It scavenges peroxynitrite, singlet oxygen, and hydroxyl radicals, while simultaneously enhancing the activity of endogenous antioxidant enzymes [118]. Ferroptosis and cuproptosis are two separate kinds of metal-dependent controlled cell death that melatonin directly influences during reproduction. Melatonin supplementation (2 mg/day for ≥8 weeks) significantly improved IVF results, especially for women aged ≥38 years, with clinical pregnancy rates more than twice those of the control group in a prospective study including AMA women [119]. Melatonin restored mitochondrial membrane potential, reduced lipid peroxidation, and elevated GPX4 expression in cumulus and granulosa cells. It also altered genes associated with cuproptosis, including ATP7B, which regulates copper export. This indicates that it has a more extensive function in safeguarding cells against metal-induced mortality [120]. In HGL5 granulosa cells, melatonin served two significant functions: it enhanced mitochondrial respiration, restored glycolysis and TCA cycle flow to baseline levels, and reduced mitochondrial ROS. Melatonin augments ferroptosis resistance by activating Nrf2, which in turn stimulates the transcription of GPX4 and SLC7A11 [119]. Melatonin protects granulosa cells against two intersecting apoptotic pathways, while simultaneously decreasing labile copper concentrations and blocking abnormal protein lipoylation that results in cuproptosis. This delineates melatonin, from a translational perspective, as a specific adjuvant that safeguards oocytes against ferroptotic and cuproptotic injury, rather than as a general antioxidant [119]. This is particularly significant for women over 35, whose oocytes exhibit indications of elevated oxidative stress and mitochondrial impairment. Melatonin’s clinically proven improvement in live birth rates in women receiving therapy offers strong rationale for its use into ART, possibly serving as a cost-effective, safe, and physiologically suitable adjuvant.

While GPX4 was formerly regarded as the only regulator of ferroptosis, increasing evidence suggests that ferroptosis may transpire even with functional GPX4, revealing other mechanisms of cell death. GPX4 utilises GSH as a cofactor to convert phospholipid hydroperoxides (PLOOHs) into their corresponding alcohols (PLOHs) [14]. Should it cease, lipid peroxidation and cellular apoptosis occur. Ma et al. (2022) examined GPX4-independent mechanisms of ferroptosis, encompassing the GCH1–BH4 axis that produces the robust lipid antioxidant tetrahydrobiopterin [113]. The DHODH pathway in mitochondria, which sustains ubiquinone reduction within the inner mitochondrial membrane; and the FSP1–CoQ10–NADH pathway, which transforms ubiquinone into ubiquinol, a radical-trapping antioxidant that prevents the propagation of lipid peroxides at the plasma membrane [121]. Suppression of ferroptosis is superfluous and compartmentalized, providing protection across organelles, as shown by these concurrent systems. This redundancy in ovarian biology likely explains patient variability: whereas some women’s oocytes may be protected by FSP1 or DHODH activity, others may rely more on GPX4, affecting their responses to chemotherapy, aging, or oxidative stress [122]. This broadens the range of possible treatment strategies beyond GPX4 stabilization. For example, DHODH activators or inhibitors may modulate mitochondrial susceptibility, while CoQ10 supplementation enhances mitochondrial bioenergetics and FSP1-mediated ferroptosis inhibition. Additionally, BH4 supplementation or modification of GCH1 activity may provide enhanced protection against ferroptosis in reproductive cells [123]. Importantly, targeted targeting is facilitated by a comprehension of these superfluous defenses: Enhancing GPX4 and FSP1 may safeguard granulosa cells and oocytes, whilst inhibiting these pathways in ectopic endometrial stromal cells may promote lesion regression. Thus, GPX4-independent pathways provide a new treatment approach to balance illness prevention with the maintenance of fertility.

FSP1 revolutionised the field by demonstrating a lipid peroxidation resistance mechanism independent of GPX4. FSP1, a flavoprotein oxidoreductase, localises to the plasma membrane via N-myristoylation, using NADH to convert oxidised ubiquinone into CoQ10H_2_ [124]. Ubiquinol is a lipophilic RTA that stabilises membranes and prevents the occurrence of lipid peroxidation via a chain reaction. Yang et al. (2022) assert that FSP1 is crucial for retinal pigment epithelial cells; its overexpression conferred complete resistance to oxidative damage, while its absence led to severe lipid peroxidation and cell death [125]. In ovarian biology, FSP1 presumably has a similar protective function in granulosa and cumulus cells, where the plasma and mitochondrial membranes are rich in peroxidation-sensitive polyunsaturated fatty acids (PUFAs) [126]. CoQ10 supplementation, often used in IVF to mitigate reduced ovarian reserve, may provide two benefits: (i) augmenting mitochondrial ATP production and (ii) strengthening the FSP1–CoQ10–NADH ferroptosis defensive mechanism. This dual mechanism may explain why CoQ10 enhances oocyte quality and embryonic development, especially in older women [127]. The FSP1 axis offers potential for treatment sensitisation; blocking FSP1 in chemoresistant tumours or ectopic endometriotic stromal cells may reveal ferroptosis sensitivity in a targeted fashion while maintaining gamete functionality. The dual therapeutic approach of ferroptosis in reproduction is exemplified by the FSP1–CoQ10–NADH pathway, which entails enhancing FSP1 activity in oocytes and granulosa cells to preserve fertility while decreasing it in diseased tissues to promote disease regression. Table 3 enumerates potential therapies that modulate ferroptosis and redox homeostasis within the ovarian microenvironment. It enumerates the processes, ovarian targets, amount of proof, and critical aspects necessary to translate the molecular framework into effective therapies.

Table 3: An overview of potential therapeutic interventions that modify redox biology and ferroptosis within the ovarian follicular milieu. The treatments include direct ferroptosis inhibitors, iron chelation, mitochondrial and antioxidant support, transcriptional stimulation of endogenous defenses (Nrf2), and cell-based exosome therapies. Evidence is obtained from early human cohorts, as well as animal and in vitro models.

The regulation of ferroptosis, as seen in Table 3, is multifaceted and modular. This indicates that it may focus on downstream effectors (lipid RTAs, GPX4/FSP1 support) and upstream drivers (iron management, NOX) depending on the circumstances. Melatonin is unique in advanced maternal age with both clinical signs and molecular complexity (Nrf2-GPX4-xCT activation, mitochondrial rescue). Ferrostatin-1/liproxstatin-1 establishes the pharmacological limit for ferroptosis inhibition in preclinical investigations, whereas CoQ10 and vitamin E promote the FSP1–CoQ10 radical-trapping and lipid-peroxidation suppression, respectively. Chelators (DFO) and the inhibition of ferritinophagy are justified in scenarios marked by iron excess (endometriosis, chemotherapy-induced). Exosome treatments and Nrf2 activation safeguard granulosa cells against chemotherapy-induced POI. Ferroptosis in endometriosis adversely affects gametes but is advantageous for combating lesions, requiring targeted approaches that inhibit ATF4 xCT in lesions while safeguarding follicles. In conclusion, the table delineates a paradigm for precision-redox therapies that may be classified by FF biomarker profiles and assessed prospectively.

The treatment landscape for ferroptosis in reproduction is rapidly evolving. It currently encompasses a range from pathway-specific precision modulators to broad-spectrum antioxidants. Conventional antioxidants such as vitamin E, NAC, and melatonin safeguard individuals globally by eliminating free radicals, replenishing GSH, and enhancing mitochondrial function [128]. However, it is now evident that non-specific scavenging has constraints: excessive consumption of antioxidants may obstruct physiological ROS signaling, which is essential for early embryonic development, fertilization, and ovulation. Consequently, the subsequent frontier is precise modulation [129]. Melatonin, sulforaphane, and bardoxolone methyl are Nrf2 activators that enhance endogenous antioxidant gene networks via an upstream technique. In oocytes, GPX4 stabilisers provide direct protection against lipid peroxidation. Iron chelators (deferoxamine, deferiprone) diminish catalytic Fe^2+^ reservoirs that facilitate the Fenton reaction, while FSP1 activators or CoQ10 supplements enhance membrane protection [130]. In intricate environments, exosomes generated from stem cells, which include proteins and microRNAs that activate Nrf2 and GPX4, have shown potential in preclinical models of POI, protecting the ovarian reserve against chemotherapy-induced ferroptosis [131]. Importantly, disease-specific customisation is imperative: Ferroptosis inhibitors enhance IVF outcomes and rejuvenate follicular health in PCOS and ageing; the stimulation of ferroptosis in stromal lesions may lead to the regression of ectopic tissue in endometriosis, while antioxidants concurrently safeguard oocytes [63]. This underscores the need for dual-modality regimens that include ferroptosis sensitization in diseased tissues and ferroptosis suppression in gametes. In the future, patient stratification will be facilitated by integrating genetic polymorphisms (e.g., GPX4, FSP1 variations, and Nrf2 activity) and FF redox biomarkers (ORP, GSH, 8-OHdG, MDA) into clinical trial design. This will ensure that treatments are provided to those most likely to benefit. The major objective is to preserve fertility while tackling underlying reproductive disorders by shifting from empirical antioxidant supplements to precision ferroptosis treatment in IVF.

## 5. Discussion

A trio of evidence redox imbalance, iron dysregulation, and lipid peroxidation illustrates that ferroptosis is a context-dependent element influencing oocyte survival and the results of ART. Studies of human FF in PCOS consistently reveal low-to-moderate mechanistic indicators of ferroptosis (xCT/GPX4 axis), increased oxidative damage markers (e.g., MDA, 8-OHdG), and altered antioxidant capacity/oxidation-reduction potential. These findings provide modest overall support and are consistently linked to reduced fertility and embryo quality. While first human data on tissue and follicular fluid are becoming available, preclinical studies indicate that ferroptotic cell death occurs in ovarian cells, with subsequent restoration achieved with the use of ferroptosis inhibitors in chemotherapy-induced POI. These data combined provide substantial (preclinical) to moderate (clinical) evidence that ferroptosis contributes to follicle loss and diminished ovarian reserve. Due to the ongoing inconsistency in human FF/ART data, we examine fresh evidence linking ferroptosis to decreased MII yield and competence in a physiologically coherent manner. In aging/DOR, iron buildup, membrane PUFA remodelling, mitochondrial ROS, and the proposed reduction in GPX4 form a cohesive process. Iron-rich retrograde menstruation and exposure to heme and haemoglobin foster a pro-lipid-peroxidation milieu in endometriosis; moderate to strong participation is shown by persistently raised human follicular fluid markers and indicators of oocyte/embryo performance. Effect sizes are negligible in contexts marked by substantial inflammatory/metabolic confounding and peak in circumstances requiring direct iron. In clinical practice, these gradients emphasise uniform FF panels for assessing ART prognosis and align with tested interventions.

Our analysis highlights ferroptosis as a significant molecular mechanism linking several ovarian pathologies, such as PCOS, POI, ovarian aging, and endometriosis, to oxidative stress, iron excess, lipid peroxidation, and granulosa cell failure. Integrating data from in vitro tests, animal models, and human investigations provides a comprehensive understanding. Ovarian cells are vulnerable to ferroptotic death owing to dysregulated iron and lipid metabolism, coupled with impaired redox defenses, which diminish oocyte competence, embryo quality, and ultimately, IVF success. Antioxidant protective networks, particularly those controlled by Nrf2, FSP1, and GPX4, provide concurrent therapeutic targets. Ferroptosis is thus a therapeutic target for fertility preservation and optimization of assisted reproductive technology, while also posing a danger to reproductive potential. Table 4 lists the main diseases linked to ferroptosis in reproductive medicine, along with the clinical or experimental models used, the important molecular mechanisms found, and the possible treatment options being looked into.

Table 4: Evidence linking ferroptosis to ovarian disorders. The table consolidates significant experimental models, molecular pathways, ovarian/follicular results, and proposed treatments for endometriosis, PCOS, chemotherapy-induced POI, and ovarian aging.

Ferroptosis has a uniform pattern marked by iron accumulation, GPX4 inhibition, and lipid peroxidation in diverse ovarian illnesses, as shown in Table 4, yet, each condition features unique regulatory mechanisms. In chemotherapy-induced primary ovarian insufficiency, the transcriptional increase in ACSL4 and the iron release mediated by HO-1 are significant. In PCOS, androgen-induced ferritinophagy and ncRNA-mediated regulation are of greater significance. Ferroptosis is associated with mitochondrial breakdown and YAP signaling in ovarian aging. In endometriosis, a paradoxical equilibrium exists: granulosa cells undergo ferroptosis owing to iron-laden follicular fluid, whilst ectopic stromal cells persist by elevating ATF4-xCT levels. The mechanistic distinctions provide a framework for the focused development of therapies in reproductive medicine, including customized approaches such as ferroptosis inhibitors, redox-modulating supplements, and stem cell-derived exosomes.

Li et al. presented solid data about human PCOS, illustrating that androgen exposure triggers NCOA4-dependent ferritinophagy in granulosa cells, resulting in the depletion of ferritin reserves, the release of labile iron, and the onset of lipid peroxidation cascades [40]. In a DHEA rat model, the ferroptosis inhibitor ferrostatin-1 ameliorated polycystic morphology and anovulation, demonstrating a clear relationship between ferroptosis suppression and the restoration of fertility [40]. Huang et al. substantiated these results by identifying ferroptosis-associated differentially expressed genes (DEGs) (ATF3, BNIP3, DDIT4, LPIN1, NOS2, NQO1, SLC2A1, SLC2A6) that are prominent in ROS metabolism and antioxidant pathways in PCOS granulosa cells [50]. The clinical importance of some of these genes was highlighted by their correlation with diminished fertilization and embryo quality. Zhang et al. significantly increased regulatory complexity by demonstrating that circRHBG promotes granulosa cell proliferation in PCOS via competitive binding to miR-515/SLC7A11 and the suppression of ferroptosis [130]. This indicates that noncoding RNAs may modulate susceptibility to ferroptosis, functioning as both therapeutic targets and diagnostic markers. Lingzhi Zhang et al. reinforce the notion that iron overload serves as a principal pathogenic mechanism by illustrating that transferrin receptor-mediated iron uptake activates NOX1/PINK1/ACSL4 signalling, resulting in mitochondrial ROS production, lipid peroxidation, and mitophagy in granulosa-like cells [130]. These findings together define PCOS as a condition marked by iron dysregulation, elevated androgen levels, and an aberrant redox balance, which together precipitate ferroptosis, negatively impacting follicle health and IVF results [132].

There is substantial data indicating that chemotherapy may harm the ovaries. Chen et al. contend that cyclophosphamide stimulates heme catabolism and the liberation of free iron via the overexpression of HO-1 [122]. This therefore results in the accumulation of ROS in mitochondria and the depletion of GPX4 in granulosa cells. HO-1 may facilitate ferroptosis and serve as an indicator of stress. Wang et al. presented supplementary data on ovaries subjected to cisplatin treatment, revealing that membranes were prepared for peroxidation via SP1-dependent transcriptional activation of ACSL4. Ferrostatin-1 and rosiglitazone, an ACSL4 inhibitor, showed potential as therapeutic agents by mitigating ovarian damage and revitalizing follicles [112]. Du et al. established that cisplatin causes ferroptosis and ovarian fibrosis via the modulation of ACSL4, ALOX15, SLC7A11, and GPX4 expression [133]. Translational investigations by Zhou et al. and Dai et al. elucidated molecular insights, revealing that exosomes from mesenchymal stem cells suppress ferritinophagy and activate Nrf2/GPX4 to alleviate ferroptosis in chemotherapy-induced POI, respectively [22,131]. Pan et al. demonstrated the adaptability of stem-cell-based approaches by revealing that endometrial stem cells function similarly via Nrf2 overexpression [72]. All of these findings indicate that inhibiting ferroptosis may assist cancer patients in preserving their fertility, and they further demonstrate that ferroptosis is central to chemotherapy-induced ovarian insufficiency.

The correlation between ferroptosis and ovarian aging has only lately been investigated. Jia et al. found more than 6000 differentially expressed genes by transcriptome profiling of granulosa cells from women of advanced maternal age, emphasizing enhanced pathways in oxidation-reduction, mitochondrial activity, and ferroptosis [37]. The data indicate that ferroptosis contributes to reduced ovarian reserve and impaired oocyte competency, even in the absence of apparent disease. Lin et al. expanded upon these discoveries by using single-cell RNA sequencing and spatial transcriptomics to demonstrate the influence of ferroptosis-related genes on energy consumption in aging germ cells [134]. Their interventional arm notably indicated that treatment with DHEA, CoQ10, and Cleo-20 T3 improved IVF results by decreasing ferroptosis markers (TFRC, NCOA4, SLC3A2) and increasing GPX4 expression. This indicates that dietary modification is a viable treatment approach for ferroptosis [135]. Wang et al. provided persuasive genetic evidence that BNC1 loss triggers primary ovarian insufficiency in oocytes via NF2-YAP-mediated ferroptosis [121]. The pharmacological suppression of YAP or ferroptosis maintained ovarian reserve, demonstrating the interplay between developmental signalling pathways and ferroptosis control. The data suggest that ferroptosis functions as a mechanism connecting ovarian ageing, reduced reserve, and clinical infertility.

In the particular situation of endometriosis, ferroptosis fulfils two roles. Ni et al. discovered that vitamin E and deferoxamine helped mitigate the effects of iron-saturated follicular fluid in women with endometriosis [41]. The iron excess induces the death of granulosa cells via NCOA4-mediated ferritinophagy, resulting in slower oocyte maturation and reduced IVF success rates. Dong et al. contend that ectopic stromal cells experience autophagy-dependent ferroptosis when subjected to iron overload; yet, they may evade cell death by upregulating ATF4–xCT, signifying a survival advantage in iron-rich conditions [42]. Zhang et al. revealed that iron-induced ferroptosis facilitates fibrosis in ovarian endometriosis by redistributing stromal cell populations from mesenchymal progenitors to myofibroblasts [130]. Li et al. assert that ferroptosis is detrimental to granulosa cells and oocytes, however it may be beneficial if it occurs just in ectopic lesions [116]. In evaluating ferroptosis-modulating therapeutics for infertility linked to endometriosis, this duality highlights the need of cell-specific targeting—protecting gametes while sensitising ectopic endometrial tissue.

Multiple therapy techniques are being developed. Zhang et al. demonstrated that melatonin mitigates PM2.5-induced ovarian ferroptosis by enhancing ovarian morphology, reinstating GPX4 and xCT, and activating Nrf2 [130]. Tsui et al. clinically shown that melatonin supplementation dramatically improved IVF pregnancy and live birth rates in women of advanced maternal age, possibly via modulating genes related to ferroptosis and cuproptosis, as well as mitochondrial metabolism [119]. Yang et al. established in retinal models that both the GPX4 and FSP1 pathways are crucial for inhibiting lipid peroxidation, whereas Ma et al. investigated GPX4-independent ferroptosis, highlighting the significance of the FSP1–CoQ10–NAD(P)H route as an auxiliary defensive mechanism [113,125]. These data clearly demonstrate the translational potential of ferroptosis inhibitors and antioxidants, not as general treatments but as targeted adjuvants customized for particular ovarian diseases.

Numerous limitations persist, despite these advancements. Human clinical studies on ferroptosis are few; the majority of data are from animal or cellular models. Biomarker variability across research hinders clinical extrapolation and meta-analysis. Most research examine a single temporal snapshot rather than tracking the process longitudinally, resulting in limited understanding of the spatiotemporal dynamics of ferroptosis inside ovarian follicles. Furthermore, there exists a paucity of understanding of the genetic diversity of ferroptosis regulators among human reproductive cohorts, including polymorphisms in GPX4, FSP1, Nrf2, and ACSL4. Finally, because to the fragility of gametes and embryos, it is essential to meticulously assess the safety, dose, and long-term ramifications of ferroptosis-modulating therapies in women.

Ferroptosis is a critical biological pathway that leads to ovarian malfunction and infertility, directly impacting the effectiveness of IVF. This study highlights the dual character of ferroptosis via molecular processes and clinical observations: it may be harmful if uncontrolled in granulosa cells and oocytes, but it may have a therapeutic effect when deliberately produced in pathological conditions such as endometriosis. The principal aims for forthcoming research should include the standardization of biomarkers, the classification of patients according to redox state, and rigorously structured clinical trials evaluating ferroptosis modulators as adjuncts in ART. If successful, ferroptosis regulation might transform reproductive medicine by providing physicians with novel methods to enhance oocyte quality, optimize IVF outcomes, and preserve fertility in at-risk women.

Proposed a clinical panel of FF biomarkers and a strategy for standardisation. We propose a stratified FF panel including a core trio of global redox potential (ORP), antioxidant capacity (GSH), and DNA oxidation markers (8-OHdG) to facilitate the application of redox and ferroptotic biology. A more extensive collection is available for centres with advanced capabilities, such as lipid peroxidation (MDA or 8-isoprostane) and iron load/handling (e.g., ferritin or transferrin saturation). The expanded framework refines risk attribution concerning ferroptotic stress, while the core encompasses supplementary elements, including defensive capacity, systemic redox status, and molecular damage. A composite score (z-standardized components; higher indicates more risk) may be produced and evaluated by examining fertilisation, embryo quality, and live birth outcomes.

Procedures for standardising from pre-analytical to analytical phases: Document the patient’s age, BMI, AMH/AFC, stimulation technique, trigger type/timing, follicle size, and laterality for patient/context control. To prevent blood contamination, discard FF that is obviously hemolyzed or establish a threshold for the haemolysis index. Collection: Prioritise the first clear aspirate, aspirate FF before to flushing, and monitor the duration between retrieval and processing. Handling: Maintain on ice; partition immediately to minimise freeze–thaw cycles; clear at 4 °C (about 1500–2000 g, for about 10 min). Storage: Utilise tubes that remain unbound and can withstand freezing at −80 °C for a duration of 30 to 60 min. Record the duration required for freezing. Assays and calibration: Employ validated methodologies (e.g., enzymatic/colorimetric GSH; LC/ELISA or HPLC-ECD for 8-OHdG; LC–MS/ELISA for 8-isoprostane or standardised TBARS for MDA; immunoassay for ferritin; potentiometric ORP); conduct duplicates with internal controls, participate in external quality assessment, and document intra/inter-assay coefficients of variation (CVs). Establish guidelines for outliers and haemolysis in advance; modify models for BMI, age, procedure, and oocyte rank; and provide findings per millilitre and, in sensitivity analyses, per total protein. Validation: use internal validation techniques (bootstrap/cross-validation) and, ideally, prospective, protocol-compliant cohorts to ascertain decision thresholds using ROC analysis. A core panel (ORP + GSH + 8-OHdG) provides a modest but valuable screening. Centres may additionally include MDA/8-isoprostane and iron data, if feasible. Low-risk profiles facilitate routine management, while high-risk composite scores prioritise counselling and advocate for enhanced procedures, such as regulating lipid peroxidation, managing iron levels, and offering antioxidant support. This methodology facilitates incremental adoption and inter-center comparison without excessively taxing laboratory resources.

## 6. Conclusions

Ferroptosis has emerged as a vital redox-dependent process of cell death, significantly impacting ovarian biology and reproductive medicine. This review consolidates evidence indicating that disruptions in iron metabolism, lipid peroxidation, and antioxidant defenses collectively induce ferroptosis in granulosa cells and oocytes, negatively affecting follicular development, diminishing oocyte competence, and undermining IVF outcomes. Ferroptosis regularly functions as a cohesive mechanism connecting redox imbalance to reproductive failure in situations including polycystic ovary syndrome, chemotherapy-induced primary ovarian insufficiency, ovarian aging, and endometriosis.

The downregulation of GPX4, elevation of ACSL4, NCOA4-mediated ferritinophagy, and mitochondrial dysfunction constitute a shared pathological hallmark. Conversely, the Nrf2–xCT–GSH axis, the FSP1–CoQ10–NAD(P)H route, and exosome-mediated protection suggest compensatory mechanisms with therapeutic promise. Translational studies indicate that the suppression of ferroptosis, via small molecules such as ferrostatin-1, nutritional strategies including melatonin and CoQ10, or sophisticated biological techniques like stem-cell-derived exosomes, can alleviate ovarian damage and enhance functional outcomes in preclinical models. Clinical data increasingly indicates that the modification of redox balance in follicular fluid is associated with enhanced fertilization, embryo development, and pregnancy rates IVF cycles.

Nonetheless, considerable obstacles persist. Few human clinical studies investigate ferroptosis in reproduction, and the variability of biomarkers complicates the comparison of existing data. Ferroptosis serves dual functions: it may be detrimental to granulosa cells and oocytes, while potentially beneficial for compromised tissues such as endometriotic lesions. This implies that tactics must be customized for each cell type and circumstance. Furthermore, the long-term safety and effectiveness of ferroptosis modulators in women of reproductive age remain mostly unexplored.

In the future, including ferroptosis indicators into clinical IVF treatment may enhance precision medicine approaches by identifying women who are most likely to benefit from redox-modulating medicines. Thorough, prospective studies are crucial to validate promising biomarkers such as glutathione, malondialdehyde, ORP, and GPX4 activity, as well as to evaluate targeted medications in specifically defined patient populations, including women with PCOS, reduced ovarian reserve, and endometriosis. Ferroptosis presents both a mechanistic difficulty and a therapeutic opportunity; understanding and using this mechanism might enhance fertility preservation, maximise assisted reproductive technology, and prolong reproductive life.

## Figures and Tables

**Table 1:** Key molecular regulators and signaling pathways of ferroptosis relevant to ovarian dysfunction.

Regulator/Pathway	Molecular Role	Evidence in Ovarian Context	Functional Consequences
GPX4 (Glutathione peroxidase 4), [12,29,37]	Detoxifies lipid hydroperoxides; central ferroptosis suppressor	Downregulated in PCOS, chemotherapy-induced POI, endometriosis, ovarian aging	Loss of GPX4 increases lipid peroxidation, impairs granulosa cell survival, reduces oocyte competence
ACSL4 (Acyl-CoA synthetase long-chain family member 4), [17,38,39]	Incorporates ω-6 PUFAs into phospholipids, increasing susceptibility to peroxidation	Upregulated in cisplatin-treated ovaries, PCOS granulosa cells, endometriosis stromal cells	Promotes ferroptosis and ovarian fibrosis; inhibition by rosiglitazone restores follicular viability
NCOA4-mediated ferritinophagy, [40,41]	Releases stored iron from ferritin, increasing labile Fe^2+^ pool	Activated by androgens in PCOS, by iron-overloaded FF in endometriosis	Iron overload drives ROS accumulation, mitochondrial damage, granulosa cell ferroptosis
HO-1 (Heme oxygenase-1), [29]	Catabolizes heme, releasing free iron	Upregulated in cyclophosphamide-exposed granulosa cells	Promotes iron overload and ferroptosis in POI models
Nrf2/xCT (SLC7A11), [35,42,43]	Master antioxidant regulator; imports cystine for GSH synthesis	Activated by melatonin, MSC-derived exosomes; upregulated in resistant endometriotic lesions	Protects granulosa cells from ferroptosis; confers lesion survival in EM
FSP1–CoQ10–NAD(P)H axis, [24,44]	Parallel antioxidant system reducing lipid peroxyl radicals	Indirect evidence in ovarian aging (CoQ10 supplementation improves IVF outcomes)	Enhances resilience of granulosa and cumulus cells, improves mitochondrial function
BNC1/NF2-YAP pathway, [38]	Transcriptional regulation of oocyte lipid metabolism and redox homeostasis	BNC1 deficiency in oocytes triggers ferroptosis via NF2-YAP	Leads to premature follicle depletion and POI

**Table 2:** Follicular fluid oxidative stress biomarkers and their associations with IVF outcomes.

Biomarker	Molecular/Redox Role	Findings in IVF Context	Clinical Outcome Associations
Total GSH, [94]	Major antioxidant, cofactor for GPX4	↓GSH in patients with low fertilization rates and in endometriosis	Low GSH linked to reduced fertilization success
8-OHdG (8-hydroxy-2′-deoxyguanosine), [94]	Marker of oxidative DNA damage	↑8-OHdG in patients with poor fertilization and low-quality blastocysts	High 8-OHdG negatively associated with embryo development
ORP, [95]	Global indicator of redox balance	↑ORP correlates with ↓fertilization rates; higher ORP in low fertilization group (<80%)	Elevated ORP predicts poor fertilization in ICSI
MDA, [96]	Lipid peroxidation end-product	↑MDA in pregnant group; positive correlation with fertilization rate and grade 1 embryos	AUC = 0.74 for predicting pregnancy
NO, [96]	Dual role in vascular and redox signaling	↓NO in pregnant vs. non-pregnant women; lower FF NO associated with better outcome	Suggests threshold-dependent effect on oocyte competence
8-Isoprostane (8-IP), [97]	Oxidative stress lipid biomarker	↑8-IP in PCOS women, particularly those with miscarriage	Linked to adverse outcomes in PCOS-IVF cycles
TAC, [97]	Composite measure of non-enzymatic antioxidants	No clear correlation with IVF outcomes; elevated in PCOS but inconsistent	Potential as a supportive marker but not predictive
Lipid Peroxidation (MDA levels) in endometriosis, [98]	Reflects ROS-driven lipid damage	↑MDA in endometriosis FF compared to controls	Associated with reduced oocyte quality, age-dependent variations in severity
Composite oxidative stress profile (ROS, inflammatory markers, growth factors), [99,100]	Integrative view of FF redox status	PCOS patients show higher oxidative and inflammatory biomarkers compared to controls	Reinforces link between low-grade inflammation, oxidative stress, and impaired IVF outcomes

**Table 3:** Candidate therapeutic strategies targeting ferroptosis/redox homeostasis in the ovarian microenvironment: mechanisms, targets, evidence, and considerations.

Class/Agent	Primary Mechanism	Ovarian Target/Context	Evidence (Model/Outcome)	Key Considerations
Melatonin	Nrf2 activation → ↑SLC7A11/GPX4; mitochondrial ROS scavenging; stabilizes ΔΨm; modulates cuproptosis	Granulosa and cumulus cells (advanced maternal age, POI risk; environmental stress)	PM2.5 models: prevents GC ferroptosis and POI via Nrf2–GPX4–xCT; AMA cohort: ↑clinical/ongoing pregnancy and live birth	Generally safe; sufficient exposure (≥8 weeks in AMA study); avoid over-suppressing physiological ROS
Coenzyme Q10 (ubiquinol)	Bioenergetic support; FSP1–CoQ10–NAD(P)H radical-trapping at membranes	Aging/DOR; mitochondrial insufficiency	Multi-omics in AMA: ↑GPX4, ↓TFRC/NCOA4/SLC3A2 with supplementation; clinical signals for oocyte/embryo quality	Formulation (ubiquinol > ubiquinone); adherence; benefit greatest in older/low-reserve cohorts
Vitamin E/lipid RTAs (e.g., α-tocopherol; liproxstatin-1)	Lipid radical trapping; terminates peroxidation chain reactions	GC/oocyte protection; EM with iron overload	Vitamin E reverses EM-FF–induced GC ferroptosis and improves oocyte maturation (models); liproxstatin-1 protective in preclinical ferroptosis	Vitamin E widely used; liproxstatin-1 preclinical only; dose–response to avoid blunting needed signals
Ferrostatin-1	Prototypic ferroptosis inhibitor (lipid RTA)	GC protection (PCOS, POI, EM models)	Reverses anovulation/PCOS traits; protects ovary in cisplatin/cyclophosphamide and RPE models	Experimental tool; clinical translation pending
Iron chelators (deferoxamine; deferiprone)	Lower labile Fe^2+^; blunt Fenton chemistry and ferritinophagy sequelae	EM (iron-overloaded FF); chemotherapy-related iron release	Deferoxamine improves EM-related infertility models; strong rationale in iron-driven contexts	Monitor iron status; avoid anemia; consider timing/local delivery
Rosiglitazone (PPARγ agonist)	Functional ACSL4 inhibition; membrane remodeling toward less-peroxidizable lipids	Cisplatin-related POI; possibly EM fibrosis	Reduces ovarian injury and GC death in cisplatin models; mechanistic link to ACSL4	Metabolic off-targets; patient selection critical
Nrf2 activators (e.g., sulforaphane; melatonin)	KEAP1–Nrf2–ARE program: ↑xCT, ↑GPX4, ↑phase II enzymes	Chemotherapy-induced POI; environmental insults; aging	MSC/EnSC exosomes and melatonin signal via Nrf2; PM2.5 toxicity mitigated by Nrf2	Potency/PK vary; sustained activation has context-dependent effects (balance HO-1)
MSC/EnSC-derived exosomes	miRNA/protein cargo → ↑Nrf2/GPX4; ↓ferritinophagy; anti-oxidative reprogramming	Chemotherapy-induced POI; GC protection	hUC-MSC and EnSC exosomes restore ovarian function by inhibiting ferroptosis (in vivo/in vitro)	Manufacturing/standardization; regulatory pathway; promising FP adjuvant
Targeting ATF4–xCT (contextual inhibition)	Reduce cystine import in resistant lesions → sensitize to ferroptosis	Endometriotic stromal cells (ferroptosis resistance)	EM stromal resistance driven by ATF4–xCT; conceptual targeting for lesion control	Cell-type selectivity essential (protect oocytes/GCs simultaneously)
DHODH/mito-CoQ axis (modulation)	Mitochondrial ubiquinone reduction supports anti-ferroptotic tone	High mitochondrial ROS states (aging, chemotherapy)	Supported in other tissues; plausible in ovary; cooperates with GPX4/FSP1	Limited ovarian data; potential synergy with CoQ10
GCH1–BH4 support	BH4 as lipid antioxidant and enzymatic cofactor	Nitric-oxide/lipid-peroxidation balance	Preclinical ferroptosis suppression in other systems; ovarian role putative	Watch NO coupling; reproductive validation needed
NOX inhibitors	Reduce cytosolic ROS production	PCOS (TFRC→NOX1 axis); oxidative FF	In vitro PCOS models: NOX1 drives ROS/mitophagy/ferroptosis	Specificity required; avoid global ROS suppression that impairs ovulatory signaling
HO-1 fine-tuning	Limit heme-iron release when overexpressed	Cyclophosphamide-related POI	HO-1 upregulation linked to GC ferroptosis; inhibition reduces damage	Dual roles of HO-1; careful, context-dependent modulation
DHEA/metabolic cofactors (incl. Cleo-20 T3)	Support TCA/ETC; ↑GPX4 expression; redox–energy coupling	AMA/DOR	Reduced ferroptosis gene signature; improved IVF metrics (cohort)	Patient selection; endocrine context; quality sourcing
Environmental mitigation (PM2.5)	Reduce particulate-induced oxidative stress	Women at risk of POI	PM2.5 drives ovarian ferroptosis; melatonin protective	Public health plus individual measures; adjunct to medical therapy

**Table 4:** Evidence of ferroptosis involvement in ovarian pathologies: mechanisms, functional consequences, and therapeutic approaches.

Condition (Refs)	Model/Sample	Key Ferroptosis Mechanisms	Ovarian/Follicular Consequences	Proposed Therapeutic Strategies
PCOS [40,50,56]	Human granulosa cells; DHEA/DHT-induced PCOS rat models	↑Ferritinophagy via NCOA4; ↑Fe^2+^; ↑MDA; ↓GPX4; ↓FTH1; circRHBG–miR-515–SLC7A11 axis; TFRC–NOX1–PINK1–ACSL4 signaling	Granulosa-cell ferroptosis; mitochondrial damage; impaired oocyte maturation; anovulation	Ferrostatin-1; iron chelators; targeting circRHBG or SLC7A11
Chemotherapy-induced POI [22,59,67,71,72,131]	Cyclophosphamide- or cisplatin-treated mice; rat ovaries; granulosa cell lines (KGN, COV434)	↑HO-1 → iron overload; ↓GPX4; ↑ACSL4 via SP1; ↑ALOX15; lipid peroxidation; mitochondrial dysfunction	Granulosa-cell death; follicle depletion; ovarian fibrosis; reduced hormone levels	Ferrostatin-1; rosiglitazone (ACSL4 inhibition); vitamin E; MSC-/EnSC-derived exosomes (via Nrf2/GPX4)
Ovarian aging/DOR [37,38,73,119]	Granulosa cells from AMA women; transcriptomic profiling; multi-omics with supplementation	Dysregulated ferroptosis genes (TFRC, NCOA4, SLC3A2; ↓GPX4); impaired mitochondrial redox balance; BNC1 deficiency triggers ferroptosis via NF2–YAP	Accelerated follicle depletion; reduced oocyte quality; diminished ovarian reserve	DHEA; CoQ10; melatonin; ferroptosis/YAP inhibitors; antioxidant support
Endometriosis-related infertility [41,88,90,115]	Follicular fluid and stromal cells from EM patients; EM mouse models	Iron-overloaded FF induces GC ferroptosis (NCOA4-ferritinophagy); autophagy-dependent ferroptosis in ESCs; ATF4–xCT confers ferroptosis resistance; iron-induced fibrosis (ACSL4/ROS)	GC loss; impaired oocyte maturation; stromal fibrosis; reduced embryo quality	Vitamin E; deferoxamine (iron chelation); ferrostatin-1; selective ATF4–xCT inhibition in lesions

## Data Availability

No new data were created or analyzed in this study. Data sharing is not applicable to this article.

## Abbreviations

ART	assisted reproductive technology
CC	cumulus cells
E2	estradiol
FF	follicular fluid
FPN	ferroportin
FSP1	ferroptosis suppressor protein 1
GSH	glutathione
GPX4	glutathione peroxidase 4
KEAP1	Kelch-like ECH-associated protein 1
LH	luteinizing hormone
LIP	labile iron pool
LOOH	lipid hydroperoxide
LOX	lipoxygenase
MDA	malondialdehyde
Nrf2	nuclear factor erythroid 2-related factor 2
PCOS	polycystic ovary syndrome
PUFA	polyunsaturated fatty acid
ROS	reactive oxygen species
SLC7A11 (xCT)	solute carrier family 7 member 11 (cystine/glutamate antiporter)
TAC	total antioxidant capacity
TFRC	transferrin receptor
GPX	glutathione peroxidase
ORP	oxidation–reduction potential
IVF	in vitro fertilization
MII	metaphase II
FSH	follicle-stimulating hormone
AMH	anti-Müllerian hormone
